# Paracrine signals from HIV-1-infected immune cells reprogram cervical cancer pathways

**DOI:** 10.1016/j.isci.2026.116083

**Published:** 2026-06-04

**Authors:** Charles Ochieng’ Olwal, Ujjwal Rathore, Sara K. Makanani, Prashant Kaushal, Immy A. Ashley, Manisha R. Ummadi, Vincent Appiah, Alexandra Lindsey Djomkam Zune, Sophie F. Blanc, Declan M. Winters, Yennifer Delgado, Kapten Muthoka, Jacqueline M. Fabius, Manon Eckhardt, Robyn M. Kaake, Maureen Su, Oliver I. Fregoso, Judd F. Hultquist, Elkanah Omenge Orang’o, Danielle L. Swaney, George Boateng Kyei, Nevan J. Krogan, Peter Kojo Quashie, Yaw Bediako, Mehdi Bouhaddou

**Affiliations:** 1 West African Centre for Cell Biology of Infectious Pathogens (WACCBIP), College of Basic and Applied Sciences, University of Ghana, Accra, Ghana; 2 Department of Biochemistry, Cell and Molecular Biology, College of Basic and Applied Sciences, University of Ghana, Accra, Ghana; 3 Gladstone Institutes, San Francisco, CA, USA; 4 Quantitative Biosciences Institute (QBI), University of California, San Francisco, San Francisco, CA, USA; 5 Institute for Quantitative and Computational Biosciences (QCBio), University of California, Los Angeles, Los Angeles, CA, USA; 6 Department of Microbiology, Immunology, and Molecular Genetics (MIMG), University of California, Los Angeles, Los Angeles, CA, USA; 7 Molecular Biology Institute, University of California, Los Angeles, Los Angeles, CA, USA; 8 Academic Model Providing Access to Healthcare (AMPATH), Eldoret, Kenya; 9 Department of Bioengineering and Therapeutic Sciences, University of California, San Francisco, San Francisco, CA, USA; 10 Molecular, Cell and Developmental Biology, University of California, Santa Cruz, Santa Cruz, CA, USA; 11 Division of Infectious Diseases, Northwestern University Feinberg School of Medicine, Chicago, IL, USA; 12 Department of Reproductive Health, Moi University, Eldoret, Kenya; 13 Department of Obstetrics and Gynecology, Aga Khan University, Medical College, Nairobi, Kenya; 14 Noguchi Memorial Institute for Medical Research, College of Health Sciences, University of Ghana, Accra, Ghana; 15 University of Ghana Medical Centre, University of Ghana, Accra, Ghana; 16 Yemaachi Biotech, Accra, Ghana

**Keywords:** Virology, Oncology, Transcriptomics

## Abstract

Persistent infection with high-risk human papillomavirus (HR-HPV) is the primary cause of cervical cancer. Co-infection with HIV-1 increases the risk of cervical cancer progression 6-fold, despite adherence to antiretroviral therapy (ART). While chronic HIV-1 infection is known to cause inflammation, the paracrine effects of HIV-1-infected immune cells on cervical signaling remain unclear. We performed transcriptomics on cervical swabs from Kenyan women stratified by HIV-1 and cancer status, which revealed HIV-1 infection drove cancer-like gene expression in non-cancerous cervical cells. In parallel, global abundance proteomics and phosphoproteomics of cervical cancer cells exposed to HIV-1-infected human primary CD4^+^ T cell secretomes revealed altered MAPK, PI3K-AKT, cell cycle, and beta-catenin pathways. Concordantly, IRS1 was upregulated in both patient cervical samples and cultured cells. Our findings suggest HIV-1 dysregulates cervical cell signaling via paracrine mechanisms to phenocopy *PIK3CA*-activating mutations through IRS1-PI3K-AKT pathway activation. Our findings highlight IRS1 and the PI3K pathway as a potential therapeutic target for cervical cancer in women living with HIV-1.

## Highlights

• Bulk RNA-seq revealed cancer-like gene expression in normal cervix of HIV+ Kenyans
• Transcriptomes of HIV+ noncancerous cervix mirrors HIV- TCGA cervical cancer patients
• Secretome of HIV+ CD4^+^ T cells impinge on pro-cancer pathways in cervical cells
• Secretome of HIV+ CD4^+^ T cells activates AKT phosphorylation signaling in cervical cells

## Graphical abstract

**Figure:**
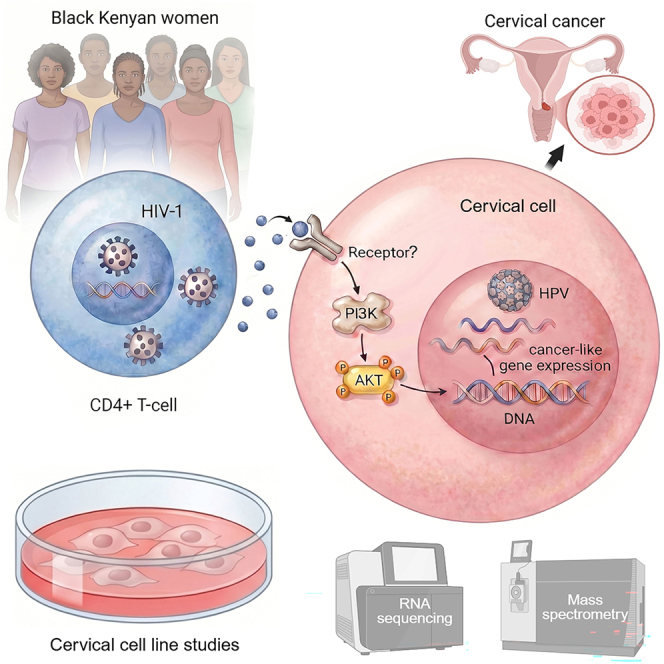

## Introduction

Cervical cancer, principally caused by high-risk human papillomavirus (HR-HPV) types, is the most frequently detected cancer in women living with HIV-1 (WLWH), especially in sub-Saharan Africa (SSA).^1^ To accelerate cervical cancer elimination, the World Health Organization (WHO) proposed a 10-year triple intervention target in 2020. The intervention encompasses: (1) 90% HPV vaccination; (2) 70% cervical screening coverage; and (3) 90% access to treatment and care for precancerous cervical lesions (PCLs) and invasive cervical carcinoma (ICC).^2^ Although massive roll-out of HPV vaccination programs in SSA could substantially reduce the incidence of cervical cancer, many young women and adolescent girls in SSA have already acquired HR-HPV^3^^,^^4^ and may not benefit from the current HPV vaccines. Furthermore, currently available HPV vaccines only protect against 9 out of 15 HR-HPV types^5^ and their efficacy against established infections is unknown.

Additionally, infection with HIV-1 is associated with accelerated progression to cervical cancer.^1^ Specifically, WLWH have a 3-fold and 6-fold elevated lifetime risk of developing PCL^6^ and progressing to ICC,^1^ respectively, compared to those who have never acquired HIV-1. Therefore, young women and adolescent girls in SSA, most of whom are highly vulnerable to HIV-1 infection,^7^^,^^8^ are at elevated risk of developing cervical cancer. In view of the shortcomings of the current HPV vaccines, and the fact that women in SSA mostly present at the late stages of cancer,^9^^,^^10^ effective treatment of ICC in the context of HIV/HPV coinfection is a critical component in the fight against this malignancy, warranting deeper investigation.

Since increased prevalence of PCL,^11^^,^^12^ and its rapid progression to ICC, have been linked with HIV-associated immune suppression (i.e., CD4^+^ T cell depletion),^13^^,^^14^ it was expected that cancer progression would decline following the advent of antiretroviral therapy (ART). Overall, prior literature suggests that accelerated progression to advanced cervical cancer in WLWH is not limited to HIV-associated immune suppression. However, studies on incidences of ICC in the ART era have yielded contradicting findings. For instance, numerous studies have reported either increased^15^^,^^16^^,^^17^^,^^18^^,^^19^^,^^20^ or reduced^13^^,^^21^^,^^22^^,^^23^^,^^24^^,^^25^^,^^26^^,^^27^^,^^28^^,^^29^ ICC incidences before and after the introduction of ART. To address this discrepancy, studies have focused on understanding the role of the immune system in ICC. To this end, some studies have suggested that low nadir,^30^ baseline,^13^ or current CD4 cell counts^27^ are the key determinants for elevated ICC risk, suggesting that immune suppression at any stage is critical for ICC development. Furthermore, a study reported that low CD4 cell count is associated with high-grade intraepithelial neoplasia related to HPV, but not cancer,^27^ implying that immune suppression may affect early stage intraepithelial neoplasia but has a minimal role on progression to ICC, which may rely on cumulative genetic changes.^31^

Several studies have reported geographical disparities on the incidences, risk, and progression of cervical lesions. Specifically, some studies have shown that residents of Africa have a tendency toward reduced lesions and regression^32^^,^^33^^,^^34^^,^^35^^,^^36^ while residents of Latin America and Asia exhibit an opposite trend^37^^,^^38^^,^^39^^,^^40^^,^^41^^,^^42^^,^^43^^,^^44^ during ART use. The geographical variations in ICC incidences persisted even after adjusting for CD4 cell counts,^45^ suggesting that factors other than the immune suppression, such as intrinsic population-specific factors or inequities in access to effective cervical cancer screening and treatment of PCL of WLWH,^45^ could be driving ICC development.

Taken together, the molecular mechanisms that underlie accelerated progression to HPV-associated cervical cancer in WLWH is not fully understood and deeper knowledge of these mechanisms could inform effective anti-cervical cancer interventions for WLWH. HPV is known to dysregulate the host signaling landscape and provoke cancer development.^46^^,^^47^^,^^48^ While HPV directly infects cervical epithelial cells, HIV-1 does not, as cervical cells lack the receptor (i.e., CD4) and co-receptors (i.e., CCR5 and CXCR4) required for HIV-1 entry. However, the cervical epithelia are in close proximity to HIV-1 target immune cells, such as CD4^+^ T cells and macrophages, which are abundant in the cervico-vaginal environment.^49^ Furthermore, HIV-1 infection is associated with heightened inflammation,^50^^,^^51^^,^^52^^,^^53^ which is associated with multiple HIV-related co-morbidities, including metabolic syndrome, neuropathic pain, chronic obstructive pulmonary disease, diabetes mellitus, among others,^54^ irrespective of ART adherence. Furthermore, compared to individuals without HIV, people living with HIV have been shown to exhibit upregulation of plasma proteins involved in inflammatory, cardiometabolic, oncologic, and neurologic processes even after long-term intake of ART.^55^

Here, we hypothesize that HIV-1 infection alters the secretome of immune cells, which dysregulates pro-cancer pathways in nearby cervical cells. A thorough understanding of how HIV-1 infection remodels the transcriptomic and proteomic landscapes of human cervical cells via paracrine mechanisms could pinpoint the molecular pathways that HIV-1 infection alters to accelerate progression to advanced HPV-associated cervical cancer. In a previous network modeling study,^56^ we highlighted the phosphatidylinositol 3-kinase-AKT (PI3K-AKT) signaling pathway as converging between HIV-1- and HPV-host protein-protein interactions, potentially underlying enhanced cervical cancer development in HPV/HIV coinfection. Here, we performed global transcriptomic profiling of cervical samples from HR-HPV-infected black Kenyan women presenting with either a normal cervix (NC; cervix without lesions), PCL or ICC who were either HIV-1 infected or uninfected. In addition, we investigated global protein abundance changes in cervical cancer cell cultures exposed to conditioned media from HIV-1-infected primary CD4^+^ T cells. Bioinformatics-based integrative analysis of our multi-omics suggested that HIV-1 infection activates and dysregulates pro-cancer signaling pathways, such as mitogen-activated protein kinase (MAPK), cell cycle, and PI3K-AKT, to accelerate HPV-associated cervical cancer development. Our findings uncover the molecular pathways that potentially underlie accelerated progression to advanced HPV-associated cervical cancer in WLWH.

## Results

### Overview of participant characteristics and study design

**Figure 1:**
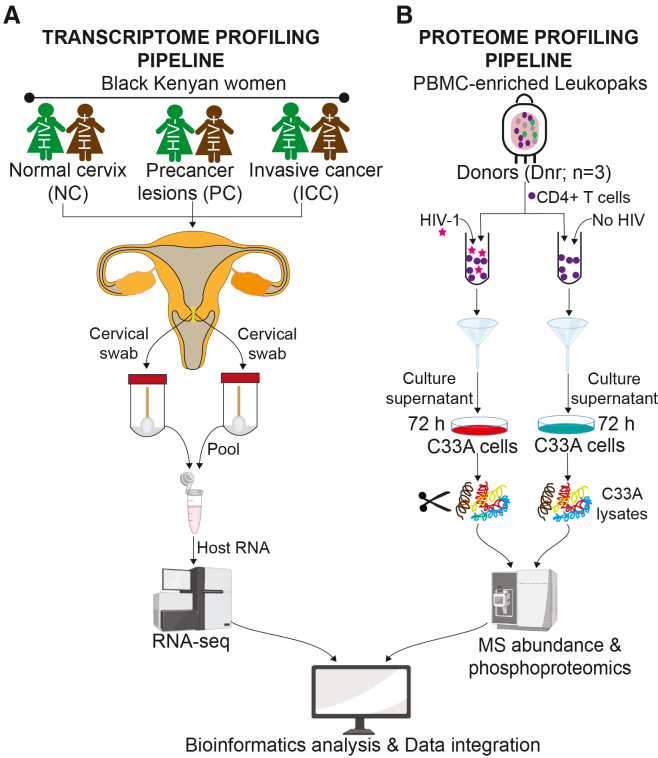
Summary of the study workflow (A) Pairs of cervical swabs were collected from Black Kenyan women stratified by HIV and cancer status. The two cervical swabs from each participant were pooled followed by PCR-based high-risk HPV testing, host RNA extraction, and bulk RNA sequencing. (B) Primary human CD4^+^ T cells isolated from PBMC-enriched leukopaks from three healthy donors (Dnr) were either infected or uninfected with a replication-competent NL4-3 HIV-1 strain for 72 h. The HIV-1-infected CD4^+^ T cell conditioned media was then used to stimulate C33A cells for 72 h. After 72 h, the C33A cell lysates were subjected to global abundance proteomics and phosphoproteomics analysis using mass spectrometry. The RNA sequencing and proteomics data were analyzed separately and integrated using bioinformatics approaches.

In this study, we set out to understand the mechanisms underlying elevated progression to HPV-associated cervical cancer seen in WLWH. First, we carried out a cross-sectional study involving black Kenyan women from western Kenya, a region with high burden of cervical cancer^57^ and HIV.^58^^,^^59^ We recruited women living with (*n* = 50) and without (*n* = 43) HIV-1 (Table S1A) who were scheduled for cervical cancer screening by visual inspection with acetic acid (VIA), a simple and cost-effective cancer screening test commonly utilized in resource-limited settings such as Kenya.^60^ Upon providing written informed consent, the participants completed a questionnaire for medical, demographic, and health histories to assess their eligibility for study enrollment (Table S1B). Prior to VIA, we collected two cervical swab samples from each participant who met the inclusion criteria (Table S1C). Based on VIA screening results, we further stratified participants according to their cancer status: NC (i.e., those with a VIA negative result), PCL (i.e., those with a VIA positive result), or ICC (i.e., those with a VIA positive or suspicious result and confirmed to have ICC by cervical biopsy). All the HIV-positive participants were on ART and had HIV viral load less than 50 copies/mL (Table S1A). For participants found to be infected with at least one of the International Agency for Research on Cancer (IARC) HR-HPV types (i.e., HPV16, HPV31, HPV33, HPV35, HPV52, HPV58, HPV18, HPV39, HPV45, HPV59, HPV51, HPV56, HPV53, HPV66, or HPV68),^61^ the two cervical swab samples collected per participant were pooled followed by host RNA extraction and bulk RNA sequencing (RNA-seq; Figure 1A).

Since the human cervico-vaginal microenvironment is composed of immune cells known to harbor HIV,^49^ we next set out to understand how the secretome of HIV-1-infected immune cells impacts the proteomic landscape of cervical cells. To this end, we cultured an HPV-negative human cervical cancer cell line (i.e., C33A^62^) with the media from primary CD4^+^ T cells infected or uninfected with a replication-competent HIV-1 NL4-3 strain (Figure S1). Briefly, bulk CD4^+^ T cells were isolated from peripheral blood mononuclear cells (PBMCs)-enriched leukapheresis products (i.e., leukopaks) from three healthy donors (Dnrs) by magnetic negative selection (i.e., to avoid activation) and challenged with HIV-1 NL4-3 for 72 h prior to the collection of conditioned media. Next, C33A cells were cultured with the conditioned media for an additional 72 h. We then quantified global changes in protein abundance and phosphorylation in the C33A cells using bottom-up mass spectrometry (MS) proteomics (Figure 1B). Finally, we performed individual and integrated bioinformatics analyses of the transcriptomic, abundance proteomics, and phosphoproteomics datasets (Figures 1A and 1B) to identify the cervical epithelial signaling pathways perturbed in the context of HIV-1 infection.

### HIV-1 infection evokes a cancer-like state in the normal cervix

**Figure 2:**
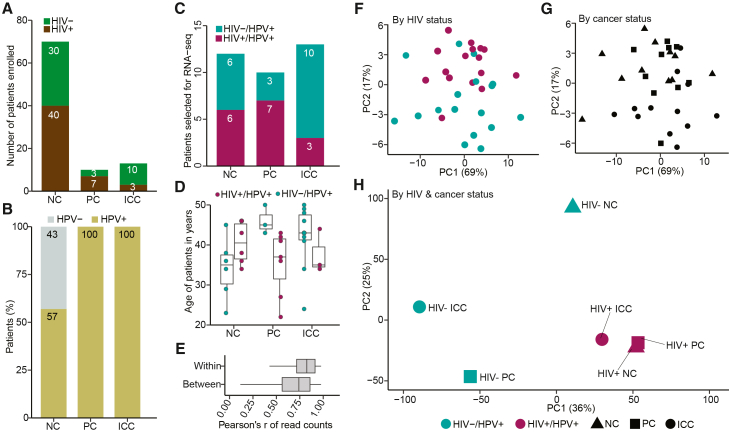
Transcriptomic profiling of cervical tissues reveals cancer-like gene expression patterns in WLWH (A) The distribution of 93 participants recruited into the study stratified based on their HIV and cancer status. (B) The distribution of high-risk HPV infection across cancer status as determined by a PCR-based genotyping technique. (C) The distribution of high-risk HPV positive participants living with and without HIV-1 across cancer status who were selected for RNA sequencing analysis. (D) The age distribution of high-risk HPV positive participants living with and without HIV-1 across cancer status selected for RNA sequencing. Each dot represents a participant. The center line in each box depicts the median age whereas the lower and upper edge of each box represent the 25th and 75th percentile values, respectively. The whiskers represent 1.5 times the interquartile range. (E) The correlation of feature counts within and between participant samples. The center line in each box represents the median whereas the lower and upper edges of each box depict the 25th and 75th percentile values, respectively. The whiskers represent 1.5 times the interquartile range. (F and G) Computational PCA plots of the feature counts from individual participants based on either their HIV or cancer status. (H) A PCA plot of feature counts averaged per HIV and cancer status.

We first sought to understand how HIV-1 infection altered the transcriptomic landscape between NC and ICC participants in the context of HR-HPV coinfection. As mentioned above, our clinical study recruited 50 women living with HIV-1 and 43 living without HIV, who were determined to either have NC, PCL, or ICC (Figure 2A). Upon testing for HR-HPV positivity, we found 57% of NC participants were HPV positive and all participants with PCL or ICC were HPV positive (Figure 2B), showing that cervical cancer in our study population is largely associated with HPV. We performed global transcriptomics (i.e., RNA-seq) analysis on cervical swabs from women who were all HR-HPV positive but split based on HIV-1 positivity (Figure 2C). The participants (*n* = 35) selected for transcriptome profiling were between the age of 22 and 50 years (Figure 2D). We noted that the median ages of WLWH with PCL (i.e., 37 years) or ICC (i.e., 35 years) were relatively lower compared to that of those without HIV and have PCL (i.e., 45 years) or ICC (i.e., 43 years) (Figure 2D), which suggest that WLWH develop precancer or advanced cervical cancer at a younger age compared to women living without HIV.

Next, we assessed whether the transcriptomic profiles could distinguish participants based on their HIV and/or cancer status. First, we decided to assess the intra- and inter-sample correlation. We observed high correlations within and between participant samples (correlation coefficients > 0.7; Figure 2E). Computational principal component analysis (PCA) of the feature counts data revealed a modest separation of individual participants with regards to HIV (Figure 2F) or cancer status (Figure 2G) along PC2. We then performed PCA on feature counts averaged across participants grouped according to HIV and cancer status. For HIV-negative women, the PCA revealed a clear segregation across cervical cancer stage (Figure 2H). Interestingly, HIV-positive women who had NC, PCL, or ICC clustered together (Figure 2H). This latter observation suggests that HIV-1 infection evokes a cancer-like state in the NC that persists and predisposes these women to PCL and ICC via dysregulated signaling mechanisms. Our observations further suggest that HIV-1 infection contributes to the entire continuum of HPV-associated cervical cancer progression, that is, from initiation to advanced stages.

### Genes upregulated in the cervix of WLWH are enriched in pro-cancer pathways

**Figure 3:**
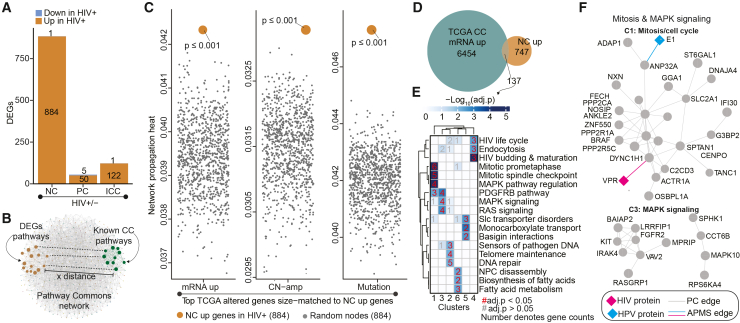
HIV-1 infection upregulates expression of genes associated with pro-cancer pathways in cervical tissues (A) Bar plots depicting the number of DEGs in WLWH relative to those without HIV across cancer status. The DEGs were based on a cutoff of |log_2_FC| > 2 and *p* < 0.05. (B) Cartoon illustrating the shared molecular networks between the DEGs and known pro-cervical cancer genes (i.e., genes commonly altered in TCGA cervical cancer Pancancer cohort) within Pathway Commons biological network. (C) Jitter plots comparing the median network propagation heats for the 884 genes upregulated in WLWH with NC versus the median propagation heats for size-matched random nodes sampled 1,000 times from each of the network propagation outputs. Separate diffused heat network propagation of the top 884 mRNA up, CN-amplified or mutated genes from TCGA cervical cancer Pancancer cohort were performed using Pathway Commons for base network. The median propagation heat of the 884 NC upregulated genes and that of 884 random nodes sampled 1,000 times were plotted per propagation category. The *p* value of the NC up genes per propagation category was calculated by first ranking the median heats then dividing the position of the median heat of NC up genes by the number of randomizations (i.e., 1,000 times). Network propagation outputs are provided in Table S2. (D) Venn diagram showing the number of DEGs in the NC group overlapping with mRNA up genes in the TCGA cervical cancer Pancancer study cohort. (E) Heatmap showing the top three canonical pathways associated with each of the subnetwork clusters of the overlapping genes in (D) above. The subnetworks were extracted from Pathway Commons. Gene-set overrepresentation analysis was performed for the network subclusters. The *p* values were calculated by hypergeometric test with multiple hypothesis testing correction (false discovery rate; FDR). A complete set of enriched biological pathways is provided in Table S3. (F) The interconnectedness of the components of the subnetwork clusters associated with pro-cancer pathways (i.e., mitosis and MAPK signaling) is highlighted. A complete network with all the six subnetwork clusters is shown in Figure S2.

We next sought to map differentially expressed genes (DEGs) to molecular pathways, comparing them across HIV and cancer status. Our analysis revealed 884, 50, and 122 genes upregulated in the HIV-positive group, relative to the HIV-negative group, for NC, PCL, and ICC, respectively (Figure 3A). Very few genes were found to be downregulated. Intriguingly, participants with NC displayed the largest differences based on HIV status whereas a much smaller magnitude of change was observed for participants with PCL or ICC (Figure 3A). This observation further supports the idea that HIV induces a cancer-like state in normal cervical epithelial cells, which becomes less distinguishable once cancer has developed (i.e., PCL or ICC). Since NC clustered with PCL and ICC for WLWH (Figure 2H), and HIV status was associated with marked transcriptional changes within the NC group (Figure 3A), we decided to focus on delineating the pathways that were dysregulated by HIV-1 in the NC group.

To assess whether the genes upregulated in the WLWH-NC group were relevant to cervical cancer development, we used a network modeling approach to evaluate whether these genes shared molecular networks with genes from The Cancer Genome Atlas (TCGA) cervical cell carcinoma database (https://www.cbioportal.org/) that were either (1) transcriptionally upregulated (“RNA-up”; top 884 genes to match upregulated genes from our NC participant cohort), (2) copy number amplified (“CN-Amp”; *n* = 884), (3) or highly mutated (“Mutation”; *n* = 884; Figure 3B). Specifically, we used network propagation, a method that simulates heat diffusion across a molecular interaction network to assess the functional proximity between gene sets. We applied this approach using the Pathway Commons network, initiating diffused heat propagation^56^ from the three sets of genes extracted from TCGA cervical cancer database. To quantify the connectivity between our gene set and known cancer-related genes, we measured the median “heat” accumulated on genes associated with RNA upregulation (“RNA-up”), copy number amplification (“CN-Amp”), or mutation in cervical cancer TCGA datasets (“Mutation”). We then compared these values to distributions generated from 1,000 random gene sets matched for size, allowing us to calculate empirical *p* values for each gene category. Strikingly, in each scenario, the set of 884 genes extracted from our patient cohort were highly ranked relative to the random sets of genes (Figure 3C), suggesting that genes upregulated by HIV-1 in NC individuals share molecular networks with genes that are commonly altered in cervical cancer patients.

Next, we sought to hone into the molecular pathways shared between the participants in our study and those from cervical cancer patients in the TCGA database. We identified 137 genes that directly overlapped between RNA-up genes (*n* = 6,455) from TCGA cervical cancer patients and from the NC group in this study (*n* = 884; Figure 3D). We extracted an interconnected subnetwork of these 137 genes from the Pathway Commons network and performed hierarchical clustering to identify six highly interconnected sub-clusters (Figure S2). A gene set overrepresentation analysis (GSOA)^56^ using canonical pathway terms downloaded from the Molecular Signatures Database (https://www.gsea-msigdb.org/gsea/msigdb) revealed HIV-related gene pathways, endocytosis, mitosis/cell cycle, RAS/ERK, DNA damage/repair, and pathogen sensors indicative of innate immune regulation (Figure 3E). Intriguingly, closer inspection of clusters 1 and 3 revealed two genes, *ANP23A* and *DYNC1H1*, were previously known^63^^,^^64^ to physically interact with HPV E1 and HIV-1 Vpr, respectively (Figure 3F). Overall, our results suggest that HIV-1 infection dysregulates pro-cancer pathways in the lesion-free cervix.

### Secretome of HIV-1-infected immune cells regulates pro-cancer proteins in cervical cells

Since HIV-1 cannot directly infect cervical cells, we sought to evaluate how secretions from HIV-1-infected CD4^+^ T cells could reprogram the expression and signaling landscape of cervical cells. To assess this, we set up a controlled experimental system in which we quantified global abundance proteomic changes in C33A cells treated with conditioned media from primary CD4^+^ T cells infected or uninfected with HIV-1. Specifically, we acquired PBMCs from three donors, isolated CD4^+^ T cells by magnetic negative selection, infected them with NL4-3 HIV-1 for 72 h, and harvested the conditioned cell culture media. We then cultured C33A cells in the CD4^+^ T cell conditioned media for 72 h before lysing the cells and performing global MS abundance proteomics and phosphoproteomics. For abundance proteomics analysis, one of the donor’s (i.e., Dnr3) T cell secretome did not induce any statistically significant changes in the abundance of C33A proteins, hence, was excluded from visualization.

**Figure 4:**
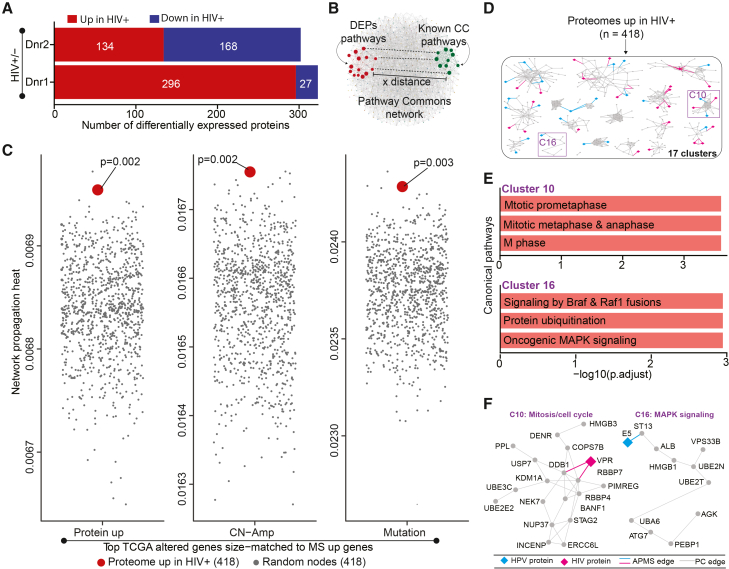
Secretome of HIV-1-infected CD4^+^ T cells upregulates proteins associated with pro-cancer pathways within cervical epithelia (A) Bar plots showing the number of differentially expressed proteins (DEP) in C33A cells stimulated with secretome of HIV-1 infected relative to uninfected primary CD4^+^ T cells. The CD4^+^ T cells were isolated from healthy donors (Dnrs). The DEPs were determined based on |log2FC| > 1 and *p* < 0.05 cutoffs. (B) Cartoon illustrating the shared molecular networks between the DEPs and known pro-cervical cancer proteins (i.e., proteins commonly altered in TCGA cervical cancer Pancancer cohort) within Pathway Commons biological network. (C) Jitter plots comparing the median network propagation heats for the 418 proteins upregulated in C33A cells treated with HIV-1-infected CD4^+^ T cell conditioned media versus the median propagation heats for size-matched random nodes sampled 1,000 times from each of the network propagation outputs. Separate diffused heat network propagation of the top proteins up (*n* = 100), CN-amplified (*n* = 418), or mutated genes (*n* = 418) from TCGA cervical cancer Pancancer cohort were performed using Pathway Commons for base network. The median propagation heat of the 418 upregulated proteins and that of 418 random nodes sampled 1,000 times were plotted per propagation category. The *p* value of the upregulated proteins per propagation category was calculated by first ranking the median heats then dividing the position of the median heat of protein up by the number of randomizations (i.e., 1,000 times). Network propagation outputs are provided in Table S4. (D) A dummy biological network generated from the upregulated proteins using Pathway Commons for base network. The network was clustered into 17 subnetworks. See Figure S4 for the actual network. (E) Bar plots highlighting the top three canonical pathways associated with clusters 10 and 16 of the network in (D). This bar plot was obtained from a GSOA performed on the subnetwork clusters from D. The *p* values were calculated by hypergeometric test with multiple hypothesis testing correction (FDR). An enrichment heatmap highlighting the top three canonical pathway terms associated with all the 17 subnetwork clusters is provided as Figure S5. A complete set of enriched biological pathways are provided in Table S5. (F) The subnetwork clusters associated with mitosis/cell cycle and MAPK signaling extracted from the bigger network in Figure S4 annotated from Pathway Commons network.

Our analysis revealed differential expression of several endogenous proteins in C33A cells treated with HIV-1-infected T cell conditioned media relative to those exposed to HIV-uninfected T cell media, with large donor-to-donor variability (Figures 4A and S3). We identified a set of 418 proteins that were upregulated in C33A cells in donor 1 or 2 and, as above, sought to assess if they were in the same network neighborhood as genes already known to be associated with cervical cancer development (Figure 4B). We extracted proteins, CN-Amp genes, or genes that were highly mutated in the TCGA cervical cancer database. Using Pathway Commons for the base network, we performed three separate network propagations: (1) top 100 upregulated proteins (*n* = 100; all the proteins upregulated in the cervical cancer TCGA cohort), (2) top 418 CN-Amp genes (*n* = 418, to match the total number of upregulated proteins from C33A cells treated with HIV-1-infected T cell conditioned media), or (3) top 418 mutated genes (*n* = 418; Figure 4B). Again, we found all three gene sets had significantly higher propagated heat relative to our randomly sampled controls (Figure 4C), suggesting that proteins upregulated by the secretome of HIV-1-infected immune cells are within overlapping network neighborhoods as the known cervical cancer genes. Furthermore, these results suggest that HIV-1 induces the secretion of proteins from immune cells that regulate the same pathways that drive cervical cancer development in HIV-1-naïve individuals.

We next sought to identify the molecular networks of proteins upregulated in C33A cells in cultured media. As above, we extracted a subnetwork of these proteins from Pathway Commons and clustered them into 17 subnetwork clusters (Figures 4D and S4). In line with our transcriptomics data, GSOA on these 17 subnetwork clusters revealed mitosis/cell cycle and MAPK signaling, especially in clusters 10 and 16, respectively (Figures 4E, 4F, and S5). Altogether, these findings suggest that the HIV-induced immune cell secretome alters pro-cancer pathways within cervical epithelia.

### HIV-1-induced paracrine signals from immune cells regulate beta-catenin, MAPK, PI3K-AKT, and cell cycle pathways

**Figure 5:**
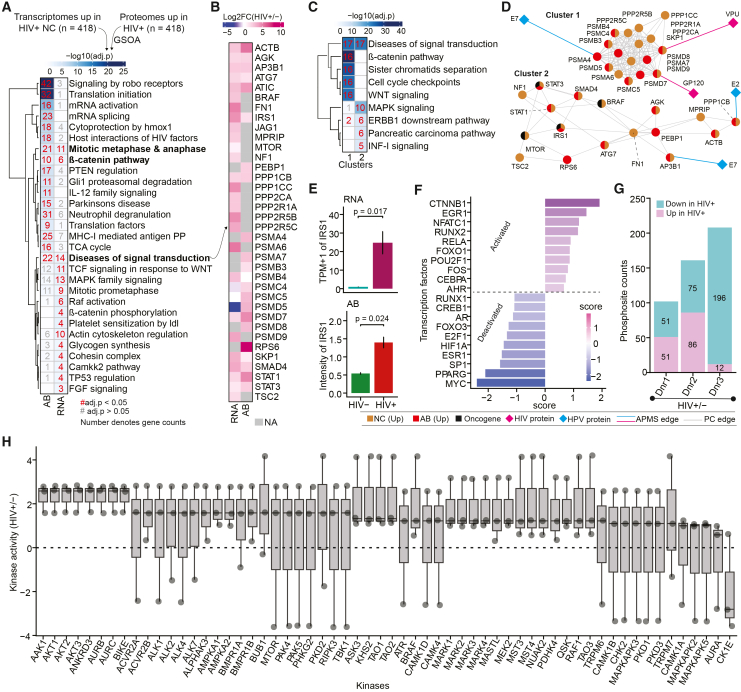
HIV-1 infection alters cell cycle and PI3K-AKT signaling pathways at the gene and protein levels (A) Heatmap highlighting the canonical pathways from GSOA of significantly upregulated proteins in C33A cells treated with HIV-1-infected CD4^+^ T cell conditioned media and size-matched upregulated genes in WLWH with NC group. The numbers depict count of proteins mapping to each canonical pathway term. Red numbers indicate a significant (adj. *p* value < 0.05) enrichment whereas gray numbers indicate a non-significant enrichment. Background color in the heatmap denotes the −log_10_ adj. *p* values as shown on color bar. The *p* values were calculated by hypergeometric test with multiple hypothesis testing correction (FDR). A complete set of enriched biological pathways are provided in Table S6. (B) A heatmap comparing the log_2_FC of genes associated with diseases of signal transduction in both the transcriptome and proteome datasets. (C) Heatmap depicting the canonical pathways from GSOA of two subnetworks annotated from Pathway Commons network using genes enriched for diseases of signal transduction in (B). The numbers depict count of proteins mapping to each canonical pathway term. Red numbers indicate a significant (adj. *p* value < 0.05) enrichment whereas gray numbers indicate a non-significant enrichment. Background color in the heatmap denotes the −log_10_ adj. *p* values as shown on color bar). The *p* values were calculated by hypergeometric test with multiple hypothesis testing correction (FDR). A complete set of enriched biological pathways are provided in Table S7. (D) Subnetworks of genes implicated in diseases of signal transduction in (B) and (C) annotated from Pathway Commons network. The HIV and HPV proteins were added based on viral-host protein-protein interactions generated in previous affinity purification MS studies.^63^^,^^64^ (E) Bar plots displaying the normalized gene expression (i.e., transcripts per million +1) and protein expression levels (mean-centered intensities) of IRS1. Statistical comparison was performed using Wilcoxon sum ranked test. Error bars depict standard error of the mean. (F) Bar plots ranking the transcription factors activated or deactivated by the genes upregulated in WLWH with NC relative to those without HIV. (G) Bar plots showing the number of differentially changing phosphosites in C33A cells treated with HIV-1 infected relative to uninfected CD4^+^ T cell conditioned media. Differentially regulated phosphosites were determined based on |log_2_FC| > 1 and *p* < 0.05 cutoffs. (H) Boxplots ranking the activities of the top regulated kinases in C33A cells exposed to HIV-1 infected relative to uninfected CD4^+^ T cell conditioned media. The top 10 kinases ranked based on the absolute kinase enrichment/activity are visualized. The center line in each box depicts the median whereas the lower and upper edges of each box represent the 25th and 75th percentile values, respectively. The whiskers represent 1.5 times the interquartile range. Each dot represents a donor.

We next sought to integrate the transcriptomics and proteomics datasets to discern if there are molecular pathways altered by HIV-1 infection at both the mRNA and protein levels. To identify pathways converging between the two datasets, we performed GSOA on the 418 proteins upregulated in C33A cells and the top 418 most upregulated mRNAs from our WLWH with NC RNA-seq dataset. Our analysis revealed convergence on three pro-cancer pathways: (1) cell cycle (i.e., mitosis), (2) the beta (β)-catenin pathway, and (3) “diseases of signal transduction” (Figure 5A). To gain a more granular picture of the “diseases of signal transduction” term, we assessed the log_2_FC of the 36 genes in this category (Figure 5B). Although some genes were upregulated in both datasets (e.g., *IRS1*, *SMAD4*, *SKP1*, and *STAT1*) others were not (e.g., *PSMD5*, *PSMA6*, and *PSMB4*). However, even though we observed examples of disjointed regulation at the gene level, we found overlapping regulation at the pathway level. We visualized the network connectivity of the 36 genes within the Pathway Commons network and clustered them into two subnetworks followed by GSOA. In alignment with the independent enrichment analysis of transcriptome and proteome datasets, GSOA revealed enrichment of cell cycle and MAPK pathways (Figure 5C). To these subnetworks, we added HIV and HPV viral proteins based on previously discovered HIV- and HPV-host protein-protein interactions from affinity purification-MS experiments^63^^,^^64^ (Figure 5C), noting several physical interactions with HIV and HPV proteins (Figure 5D), suggesting that HIV-1 and HPV proteins could directly regulate these pathways.

Interestingly, we observed that IRS1 was significantly upregulated in both the transcriptomics and proteomics datasets (Figure 5E). Increased expression of IRS1 has been associated with the activation of both MAPK and PI3K signaling pathways.^65^^,^^66^ In addition, using the decoupleR v2.12.0 package, we inferred transcription factor activities based on prior knowledge networks of transcription factor-target gene interactions.^67^ This analysis revealed the activation of CTNNB1 (β-catenin) in WLWH NC samples relative to women without HIV, which is known to be downstream of the PI3K-AKT and MAPK signaling (Figure 5F).^68^ Altogether, our results suggest that the paracrine signals from HIV-1-infected immune cells upregulate IRS1, activate the PI3K-AKT and/or the MAPK signaling pathways, and activate CTNNB1 transcription factor activity in cervical cells, which may underlie cancer progression.

The PI3K-AKT and MAPK signaling pathways are regulated by phosphorylation.^69^ To directly capture the cervical signaling pathways activated by HIV-associated paracrine signals, we performed global MS phosphoproteomics of C33A cells exposed to cultured media from HIV-1 infected or uninfected primary CD4^+^ T cells. Our analysis revealed marked remodeling of phosphorylation in treated C33A cells (Figure 5G). We next inferred kinase activities from phosphoproteomics data using The Kinase Library (v1.5.0), an algorithm of *in vitro*-derived kinase sequence motifs.^70^^,^^71^ In alignment with our transcriptomics and abundance proteomics datasets, this analysis revealed the activation of PI3K-AKT kinases AKT1, AKT2, AKT3, and cell cycle kinases AURB, AURC, CDK1, and CDK2 (Figures 5H and S6). Altogether, these findings suggest that paracrine signals from HIV-1-infected immune cells increase cell cycle progression by activating PI3K-AKT signaling in cervical epithelia.

## Discussion

How HIV-1 infection contributes to the acceleration of HPV-associated cervical cancer remains incompletely understood. The present study sought to address this question using multiple system-level approaches performed on cervical samples obtained from Black Kenyan women living with and without HIV and possessing NC, PCL, or ICC. In parallel, cervical cells in culture were stimulated with the secretome of HIV-1-infected or HIV-uninfected primary CD4^+^ T cells followed by abundance proteomics and phosphoproteomics analysis.

First, we found that all participants with PCL and ICC were HR-HPV positive, confirming that cervical cancer in our study setting is largely ascribed to HR-HPV, as expected. Furthermore, HIV-positive women with PCL or ICC had considerably lower median age relative to those without HIV. This observation is in agreement with previous reports^72^^,^^73^ and suggests that WLWH are predisposed to precancer and invasive cancer at a younger age compared to HIV-naïve women. Surprisingly, a PCA of read counts averaged per HIV and cancer status revealed that WLWH with NC, PCL, and ICC clustered together. This outcome suggests that HIV-1 infection evokes a cancer-like molecular state in HPV-infected normal cervix, which persists and predisposes women to accelerated cancer progression. Our data further support the hypothesis that HIV-1 infection contributes to the early stages of HPV-associated cervical cancer development, supported by the observation that most transcriptomic changes occurred in the HIV-positive NC group, suggesting that HIV-1 may introduce irreversible changes in cervical cells at early stages. Additionally, the clustering of HIV-positive NC, PCL, and ICC samples implies a shared HIV-driven transcriptional program across disease stages. These findings may help explain the accelerated progression to cervical cancer observed in WLWH.

The present study sought to identify the molecular pathways that are targeted by HIV-1 infection during cervical cancer development. Functional enrichment of both transcriptome and proteome data highlighted that HIV-1 infection upregulates the expression of several cancer-associated pathways in cervical cells, including MAPK, cell cycle, DNA repair, and PI3K signaling. The role of MAPK, cell cycle, DNA repair, and PI3K pathways in the development of various cancers is well documented.^74^^,^^75^ *PIK3CA*, the largest catalytic subunit of PI3K, is the second most altered gene in cervical cancer patients according to cBioPortal and has emerged as the topmost mutated gene in cervical cancer patients across ethnicities.^76^^,^^77^^,^^78^^,^^79^^,^^80^ It is noteworthy that the dysregulation of these molecular pathways occurred in the normal cervix and were also dysregulated by the secretome of HIV-1-infected CD4^+^ T cells. These findings suggest that HIV-1 infection of immune cells within the cervico-vaginal environment impacts major pro-cancer pathways in cervical cells and could explain why WLWH are prone to PCL and progression to advanced HPV-associated cervical cancer relative to HIV-naïve women. However, it remains to be determined whether the molecular pathways highlighted above are cooperatively targeted by both HIV-1 and HPV infection or not. Future studies would be needed to identify the nature of cooperation by these viral pathogens in dysregulating the pathways.

Our transcriptomics and proteomics analyses highlighted the regulation of the PI3K pathway. Specifically, we found increased IRS1 mRNA and protein expression from participant samples and our culture system, respectively, which is a known adaptor protein for PI3K pathway signal transduction from receptor tyrosine kinases.^81^ In addition, transcription factor activity analysis from the mRNA-seq data revealed the activation of CTNNB1 (β-catenin) as the top hit, which is known to be activated by PI3K signaling. Lastly, phosphoproteomics analysis of cultured cervical cells exposed to conditioned media from HIV-1-infected T cells confirmed activation of AKT, a protein immediately downstream of PIK3CA. These findings underscore the centrality of PI3K-AKT in cervical cancer development in the context of HIV-1 infection.

In addition, there is need for follow-up studies to determine the exact mechanism by which the PI3K-AKT pathway is activated, which could reveal therapeutic targets. A recent Ugandan genomics and transcriptomics study reported that mutation and mRNA expression of *PIK3CA* was lower in WLWH compared to women without HIV.^76^ This finding suggests that there is less selective pressure for *PIK3CA* mutations in WLWH. In conjunction with our findings of PI3K activation from this study, we reason that HIV-1 infection may phenocopy *PIK3CA* mutations by stimulating PI3K pathway activity through paracrine signals derived from HIV-1-infected immune cells. This concept is supported by previous studies on hepatocellular and head and neck cancers, which reported lower mutation rates of known cancer-related genes, such as *FAT1*, *CTNNB1*, *TERT*, and *TP53*, in virus-infected individuals.^82^^,^^83^^,^^84^ Future studies will be required to uncover the exact factor within the immune cell secretome that activates the PI3K pathway.

In summary, our work provides a transcriptomic resource of cervical swabs from an HIV-endemic area in SSA. Using systems biology and network modeling approaches, we highlight the key pro-cancer pathways, such as MAPK and PI3K-AKT, that may underlie enhanced progression to advanced HPV-associated cervical cancer seen in WLWH. Our findings nominate these molecular pathways for follow-up mechanistic studies aimed at identifying therapeutic targets for HPV-associated cervical cancer in WLWH.

### Limitations of the study

Our study possesses several limitations. First, although CXCR4 is widely expressed in a majority of the T cell populations, vaginal transmissions predominantly occur via CCR5-tropic HIV strains (reviewed in Duenas-Decamp et al.^85^). Future studies using CCR5-tropic virus would mirror paracrine effects in natural vaginal HIV transmissions in women. Second, our study used donors whose CD4^+^ T cells had a substantial difference in HIV-1 infection rates and elicited variability in C33A cells’ response. Validation of our findings with additional donors would strengthen our conclusions. Third, since paracrine effects were primarily assessed in HPV-negative cervical cancer cell model, future studies should aim to perform these studies in HPV-infected primary cells or animal models. Lastly, since participant data on potential confounding factors (e.g., reproductive infection history) were obtained through self-reports, it is possible there are inaccuracies.

## Resource availability

### Lead contact

Further information and requests for resources should be directed to and will be fulfilled by the lead contact, Mehdi Bouhaddou (bouhaddou@ucla.edu).

### Materials availability

This study did not generate new unique reagents.

### Data and code availability

• All raw RNA-seq data files have been deposited at NCBI’s Gene Expression Omnibus^86^ and are accessible through GEO Series accession number: GSE299125. The mass spectrometry proteomics (i.e., abundance proteomics and phosphoproteomics) data have been deposited to the ProteomeXchange Consortium via the PRIDE^87^ partner repository with the dataset identifier PXD077909. Both RNA-seq and mass spectrometry data are publicly available.
• This paper does not report original code.
• All data/images supporting the figures presented are in this manuscript and in the supplemental materials. Any additional information required to reanalyze the data reported in this paper is available from the lead contact upon request.

## Acknowledgments

We would like to acknowledge funding from the 10.13039/100000002National Institutes of Health (NIH-NIGMS R35GM160071 to M.B.), a pilot grant from the Center for AIDS Research (CFAR; 1P30AI152501-01A1 to M.B., M.S., and O.I.F.), a Collaborative Development Award (CDA) and a Mentored Scientist Award (MSA) from the HIV Accessory and Regulatory Complexes (HARC) center at the Quantitative Biosciences Institute (QBI) at 10.13039/100008069UCSF (U54AI170792 to C.O.O., M.B., M.S., and O.I.F.). In the Bouhaddou lab, our trainees were generously supported by the 10.13039/100000011Howard Hughes Medical Institute (HHMI) Gilliam Fellowship (#GT17454 to S.K.M.), Molecular Biology Institute Whitcome Fellowship to S.K.M., the UCLA-Caltech Medical Scientist Training Program (NIH-NIGMS T32GM008042 to S.F.B.), the David Geffen Medical Student Scholarship to S.F.B., the Cellular and Molecular Biology T32 at UCLA (NIH-NIGMS T32GM145388 to D.M.W.), the Microbial Pathogenesis Training Grant (MPTG NIH-NIAID T32AI007323 to Y.D.), and the Graduate Research Fellowship Program (GRFP) from the 10.13039/100000001National Science Foundation (DGE-2444110 to Y.D.). This work was additionally supported by funds from a World Bank African Centres of Excellence grant (WACCBIP+NCDs: Awandare) and a DELTAS Africa grant (DEL-22-014: Awandare) by Science for Africa Foundation to the Developing Excellence in Leadership, Training and Science in Africa (DELTAS Africa) programme with support from Wellcome and the UK Foreign, Commonwealth & Development Office and is part of the EDCPT2 programme supported by the European Union. C.O.O. was also supported by a WACCBIP-World Bank ACE PhD fellowship (WACCBIP+NCDs: Awandare). M.B. also would like to acknowledge the 10.13039/100010296James B. Pendleton Charitable Trust and the 10.13039/100002214McCarthy Family Foundation for their generous support of our research.

## Author contributions

C.O.O., conceptualization, writing – original draft, investigation, software, formal analysis, visualization, and data curation; U.R., investigation, validation, and writing – review and editing; S.K.M., investigation and writing – review and editing; P.K., investigation and writing – review and editing; I.A.A., investigation and writing – review and editing; M.R.U., investigation, validation, and writing – review and editing; V.A., software and formal analysis; A.L.DZ., investigation and data curation; S.F.B., investigation and writing – review and editing; D.M.W., data curation and writing – review and editing; Y.D., investigation and data curation; K.M., supervision and writing – review and editing; J.M.F., writing – review and editing; M.E., investigation and writing – review and editing; R.M.K., investigation and writing – review and editing; M.S., writing – review and editing; O.I.F., writing – review and editing; J.F.H., writing – review and editing; E.O.O., supervision and writing – review and editing; D.L.S., supervision, investigation, and writing – review and editing; G.B.K., supervision and writing – review and editing; N.J.K., resources, supervision, funding acquisition, and writing – review and editing; P.K.Q., resources, supervision, and writing – review and editing; Y.B., resources, supervision, funding acquisition, and writing – review and editing; M.B., software, resources, supervision, funding acquisition, and writing – review and editing.

## Declaration of interests

The Krogan Laboratory has received research support from Vir Biotechnology, F Hoffmann-La Roche, and Rezo Therapeutics. N.J.K. has a financially compensated consulting agreement with Maze Therapeutics. N.J.K. is the President and is on the Board of Directors of Rezo Therapeutics, and he is a shareholder in Tenaya Therapeutics, Maze Therapeutics, Rezo Therapeutics, and Interline Therapeutics. The Hultquist Laboratory has received research support, paid to Northwestern University, from Gilead Sciences and is a paid consultant for Merck and Ridgeback Biotherapeutics. M.B. is a financially compensated advisor for Gen1e Life Sciences and MedStat Inc.

## STAR★Methods

### Key resources table

**Table:** 

REAGENT or RESOURCE	SOURCE	IDENTIFIER
**Biological samples**		

Cervical swabs samples from Kenyans	This paper	NA
Leukopaks from healthy donors	StemCell Technologies	NA

**Critical commercial assays**		

TANBead® Nucleic Acid Extraction Kit	Taiwan Advanced Nanotech Inc.	Cat# W665S66
NEBNext® Ultra™ II RNA Library Prep Kit for Illumina®	New England Biolabs	Cat# E7770L
NEBNext rRNA Depletion Kit version (v)2	New England Biolabs	Cat# E7400×
Qubit™ 1× dsDNA High Sensitivity assay kit	Invitrogen	Cat# Q32851
EasySep™ Human CD4^+^ T cell Isolation Kit	StemCell Technologies	Cat# 17952
Human Papillomavirus DNA Diagnostic Kit	Sansure Biotech	Cat# S3057E
GenePrint® 10 system	Promega	Cat# B9510

**Deposited data**		

Raw human RNA-seq data	This paper	GEO: GSE299125
Raw mass spectrometry data	This paper	PRIDE Project ID: PXD077909

**Software and algorithms**		

R 4.5.2	R Development Core Team	https://cran.r-project.org/
FastQC v0.21.1	Andrews et al.^88^	https://github.com/s-andrews/FastQC
Cutadapt v1.18	Martin^89^	https://cutadapt.readthedocs.io/en/stable/
STAR v2.7.10b	Dobin et al.^90^	https://github.com/alexdobin/STAR/releases
Subread v2.0.6	Liao et al.^91^	https://subread.sourceforge.net/
DESeq2 v1.49.0	Love et al.^92^	https://www.bioconductor.org/packages/release/bioc/html/DESeq2.html
Spectronaut v19.8.250311.62635	Biognosys	https://biognosys.com/software/spectronaut/
DEP v1.32.0	Zhang et al.^93^	https://mirror.accum.se/mirror/bioconductor.org/packages/3.15/bioc/vignettes/DEP/inst/doc/DEP.html
limma v3.65.1	Ritchie et al.^94^	https://bioconductor.org/packages/release/bioc/html/limma.html
decoupleR v2.12.0	Badia-i-Mompe et al.^67^	https://www.bioconductor.org/packages/release/bioc/html/decoupleR.html
kinase-library v1.5.0	Johnson et al.^70^ & Yaron-Barir et al.^71^	https://kinase-library.phosphosite.org/kinase-library/
FlowJo™ v10.9	FlowJo LCC (BD)	https://www.flowjo.com
Attune™ NxT Software v3.2.0	Thermo Fisher Scientific	Cat# A25554

**Experimental models: Cell lines**		

C33A cell line	A gift from Jacques Archambault, McGill University, Canada	Auersperg^62^
HEK293T cell line	ATCC	Cat# CRL-3216

**Recombinant DNA**		

HIV-1 NL4-3 *nef*:IRES:GFP (NLENG1-IRES)	NIH AIDS Reagent Program	Cat# 11349

### Experimental model and study participant details

#### Human specimen

The study protocol was reviewed and approved by Moi Teaching and Referral Hospital/Moi University College of Health Sciences-Institutional Research and Ethics Committee (MTRH/MU-IREC; Approval # FAN: 0003853). We also obtained a research permit from the National Commission for Science, Technology and Innovation, Kenya (Approval # NACOSTI/P/21/10523). To collect samples from health facilities within western Kenya, we obtained permission from the management of the Moi Teaching and Referral Hospital (Reference # ELD/MTRH/R&P/10/2/V.2/2010). All participants provided written informed consent prior to enrolment into the study.

In this study, we recruited participants from two Academic Model Providing Access to Healthcare (AMPATH; https://www.ampathkenya.org/) clinics in Kisumu and Trans-Nzoia counties (https://www.ampathkenya.org/where-we-work) in western Kenya. In this cross-sectional study, we targeted 93 Black Kenyan women who were either HIV-positive (n = 50) or HIV-negative (n = 43) and were having NC, PCL or ICC based on a VIA test and/or cervical biopsy analysis. We confirmed the HIV status of the participants from their medical records. Only participants without syphilis infection from a VDRL test (Table S1A), any other sexually transmitted diseases and chronic illnesses (Table S1B) were eligible for sample collection. All HIV-infected participants were on ART. Prior to the VIA test performed as detailed previously^95^ by competent AMPATH nurses who were re-trained on VIA procedure every six months,^96^ two cervical swab samples were collected from every participant who consented and met the inclusion criteria (Table S1C). Each cervical swab was put into a cryovial containing 1 mL of 1X phosphate-buffered saline (PBS) and stored at -80°C until use. Those with VIA negative and VIA positive results were classified as NC and PCL, respectively. In addition to the two cervical swabs, a cervical biopsy sample was collected from women found to be suspicious for ICC for cervical biopsy test, which we used to classify the participants into ICC group. The presence of at least one of the IARC HR-HPV types^61^ in the cervical samples was assessed using Human Papillomavirus DNA Diagnostic Kit (Ref #S3057E; Sansure Biotech) following the manufacturer’s instructions.

#### Cell lines

For this study, we used C33A cells gifted by Jacques Archambault from McGill University, Canada. HIV-1 virus was produced using HEK293T cells (ATCC, Cat# CRL-3216). Both cells were maintained in Dulbecco’s Modified Eagle Medium (DMEM) supplemented with 10% fetal bovine serum (FBS, Gibco) and 1% penicillin/streptomycin (Corning). The cell lines were authenticated by Short Tandem Repeat analysis using the GenePrint® 10 system (Promega) at the University of California, Berkeley Cell Culture Facility. All the cell lines tested negative for mycoplasma contamination prior to commencement.

#### Primary human CD4^+^ T cells

Primary human CD4^+^ T cells were isolated as described previously.^97^ In brief, PBMCs were first isolated from leukopaks (leukopaks; StemCell Technologies) from healthy deidentified donors by Ficoll centrifugation using SepMate tubes following the manufacturer’s instructions (StemCell). CD4^+^ T cells were isolated from PBMCs by magnetic negative selection using an EasySep™ Human CD4^+^ T Cell Isolation Kit (StemCell, Cat# 17952) and cultured in resting-cell complete Roswell Park Memorial Institute (RPMI)-1640 medium, which was prepared by supplementing RPMI-1640 (Sigma) with 5 mM sodium pyruvate (Corning), 2 mM glutamine, 5 mM 4-(2-hydroxyethyl)-1-piperazineethanesulfonic acid (HEPES, Corning), 50 μg/mL penicillin/streptomycin (Corning), 10% FBS (Gibco), 10 IU/mL of interleukin-2 (IL-2; Miltenyi Biotec) and 5 ng/mL IL-7 (R&D Systems). The CD4^+^ T cells were activated using anti-human CD3/CD8 CTS Dynabeads (Fisher Scientific #40203D) at a 1:1 cell:bead ratio at 1 × 10^6^ cells/mL.

### Method details

#### RNA-seq of participant cervical samples

In line with our aim of understanding the role of HIV-1 in accelerating progression to HPV-associated cervical cancer, we selected participants (n = 35) who were HR-HPV positive and either living with or without HIV and had NC, PCL or ICC. For RNA-seq analysis, we pooled two cervical swab samples collected per participant. Total RNA was extracted from 300 μL of the pooled cervical samples using an automated nucleic acid extraction system (TANBead maelstrom 9600) and TANBead® Nucleic Acid Extraction Kit (Cat# W665S66) following the manufacturers’ instructions. Contaminating genomic DNA was degraded using RNase-free DNase I (Thermo Scientific) following the manufacturer’s protocol. RNA was eluted in 40 μL of elution buffer. Aliquot of the RNA (2 μL) was used for bioanalysis using Agilent TapeStation system following the manufacturer’s instructions. The remaining RNA was kept at -80C until use. NEBNext® Ultra™ II RNA Library Prep Kit for Illumina® (New England Biolabs, Cat# Q33252) together with the NEBNext rRNA Depletion Kit version (v)2 (New England Biolabs, Cat# E7400X) were used for RNA library preparation as detailed in section 2 of the product manual. Libraries were processed on a SimpliAmp™ Thermal Cycler (Thermo Fisher Scientific). Sequencing libraries were quantified using Qubit™ Flex Fluorometer and Qubit Flex Assay Tube Strips (Invitrogen, Cat# Q33252) after treatment with Qubit™ 1X dsDNA High Sensitivity assay kit (Invitrogen, Cat# Q32851) following the manufacturer’s recommendations. Subsequently, the samples were sequenced on a Nextseq 550 platform (Illumina) using Nextseq 550 High Throughput Kit (Illumina) to generate 150 base paired end reads.

#### Preparation of HIV-1 stock

In this study, a replication-competent CXCR4-tropic subtype-B pNL4-3 (NLENG1-IRES or NLENG1I) molecular clone, in which GFP is cloned behind an internal ribosomal entry site cassette following the HIV Nef gene^98^ was used. HIV stocks were prepared as described previously.^97^ In brief, 5 × 10^6^ HEK293T cells were transfected with 10 μg of the molecular clone (PolyJet, SignaGen) as per the manufacturer’s instructions. Day 2 and 3 post-transfection, 25 mL of the culture supernatant was harvested and filtered through 0.45 mm polyvinylidene fluoride (PVDF) filters (Millipore). The filtrate was precipitated in 8.5% polyethylene glycol (PEG, average M_n_ 6000, Sigma), 0.3 M sodium chloride at 4°C overnight. The supernatant was centrifuged at 3500 rotations per minute (rpm) for 20 minutes and suspended in 0.5 mL of PBS for a 100x effective concentration. Aliquots were kept at −80°C until use. The GFP-tagged virus supernatant produced from the HEK293T cells was titrated in primary human CD4^+^ T cells and the virus dilution required to achieve a target infection rate was determined using flow cytometry.

#### Infection of primary CD4^+^ T cells with HIV-1 and isolation of secretome

Isolated CD4^+^ T cells were infected with HIV-1 as described previously.^97^ In brief, 2 days post-isolation, CD4^+^ T cells were plated in round-bottom plates in triplicate and cultured overnight in 150 μL of complete RPMI-1640. The following morning, 2.5 μL of HIV-1 stock in a 50 μL carrier volume (i.e., complete RPMI) was pipetted into each well. For the HIV-uninfected wells, 50 μL of the carrier volume without the virus was pipetted into each well. The plate was spinoculated at 1200 x g for 2 hours at 22°C to enhance infection and then washed twice with 1X PBS. Complete RPMI (i.e., 200 μL) was gently pipetted into each well and cells were incubated at 37°C in 5% CO_2_ for 3 days. The plate was then centrifuged at 400 rpm for 5 minutes at room temperature. The supernatant was transferred to a fresh plate and re-centrifuged to remove any residual cells. The supernatant was stored at -80°C awaiting stimulation of cervical cells.

#### Flow cytometry analysis of primary CD4^+^ T cells post-HIV infection

The CD4^+^ T cell pellets from the preceding section were subjected to flow cytometry analysis to assess the infection rates via GFP-tagged HIV-1. In brief, the Attune NxT Acoustic Focusing Cytometer (ThermoFisher) was used to perform flow cytometric analysis on HIV-1-infected and HIV-uninfected CD4^+^ T cells. All events were recorded in a 100 μL sample volume following one 150 μL mixing cycle. The data in FCS3.0 file format were exported using Attune™ NxT Software v3.2.0 and analyzed on FlowJo™ v10.9 using a consistent template.

#### Stimulation of cervical cells with secretome of HIV-1-infected primary CD4^+^ T cells

Approximately 7.3 × 10^5^ C33A cells were plated into 10-cm dishes (Corning) and incubated at 37°C with 5% CO_2_ for 24 hours. The spent media was gently replaced with 12 mL of media containing 1:1 mixture of complete DMEM and supernatant collected from either HIV-1-infected or HIV-uninfected (controls) primary CD4^+^ T cell cultures every 24 hours. The dishes were incubated at 37°C for a total of 72 hours. For each condition (i.e., HIV-infected and HIV-uninfected), three technical plates were prepared.

#### Sample preparation for MS abundance proteomics and phosphoproteomics of cervical cells stimulated with secretome of HIV-1-infected primary CD4^+^ T cells

Upon stimulation of C33A cells with secretome of primary CD4^+^ T cells infected or uninfected with HIV-1 for 72 hours, MS analysis (i.e., abundance proteomics and phosphoproteomics) was performed on these epithelial cells as detailed previously.^99^ In brief, C33A cell samples were washed twice with cold PBS, lysed in 6 M guanidine hydrochloride (Sigma-Aldrich) and boiled at 95°C for 5 min then kept on dry ice. The lysed samples were thawed and sonicated using a probe sonicator 1x for 15 seconds at 20% amplitude. After centrifugation of the lysed samples at ∼13,000 rpm for 10 minutes, supernatant was transferred to a new protein lo-bind tube. Protein was then quantified using a Bradford Assay. About 500 μg of protein sample was processed further, starting with reduction and alkylation using a 1:10 sample volume of tris-(2-carboxyethyl) (TCEP) (10 mM final) and 2-chloroacetamide (4.4mM final) for 5 minutes at 45°C with shaking. Before digestion of protein, we diluted the 6M guanidine hydrochloride 1:6 with 100 mM Tris-HCl pH8 to enhance trypsin activity and LysC proteolytic enzymes, both of which were added at a 1:100 (wt/wt) enzyme-substrate ratio and put in a water bath 37°C overnight (∼16 hours). Thereafter, 10% trifluoroacetic acid (TFA) was added to each sample to attain a pH2.0. Desalting of the samples was done using a vacuum manifold with 50 mg Sep Pak C18 cartridges (Waters). Activation of each cartridge was done using 1 mL 80% acetonitrile (ACN)/0.1% TFA followed by equilibration with 3 × 1 mL of 0.1% TFA. After loading the samples, we washed the cartridges with 3 × 1 mL of 0.1% TFA. The samples were then eluted with 1 × 0.8 mL 50% ACN/0.25% formic acid (FA). Approximately 50 μg (10%) of the resulting volume was kept for abundance proteomics and the rest was used for and phosphoproteomics analysis. All samples were dried by vacuum centrifugation. For phosphopeptide enrichment, Ti-IMAC beads (Resyn Biosciences) were aliquoted in a 1:2 (w/w) peptide:beads ratio and equilibrated three times with binding buffer (0.1 M glycolic acid in 80% acetonitrile (ACN), 5% TFA). Dried peptide samples were resuspended in 200 μL of binding buffer, added to the equilibrated beads, and incubated for 30 min at 23°C, 1200 rpm. The unbound fraction was discarded, and beads were washed sequentially once each with 200 μL of the binding buffer, wash buffer 1 (60% ACN, 1% TFA, 200 mM NaCl), wash buffer 2 (60% ACN, 1% TFA), and finally with LC-MS grade water. Enriched phosphopeptides were eluted twice by incubating the beads with 150 μL of 1% (v/v) ammonium hydroxide (Sigma) in LC-MS grade water (Fisher Scientific) for 10 min at 23 °C, 1200 rpm. The eluted peptides were transferred to a new protein LoBind tube containing 50 μL of 10% (v/v) formic acid in LC-MS grade water. Both eluates were pooled and dried then resuspended in 0.1% formic acid for LC/MS analysis.

### Quantification and statistical analysis

#### Bioinformatics analysis of RNA-seq data

The raw RNA-seq reads were subjected to quality control using the FastQC v0.21.1^88^ and trimmed using Cutadapt v1.18^89^ to filter out poor-quality reads and adapters. After trimming, we mapped the reads to the human reference genome (hg38) curated by the University of California, Santa Cruz using STA v2.7.10b.^90^ We counted the reads per transcript using featureCount of the Subread package v2.0.6^91^ to generate a count matrix data. We loaded the count matrix data onto RStudio v2026.01.0+392 anchored in R software v4.5.2. To identify outlier samples, we generated a PCA and inter- and intra-sample correlation plots from transformed read counts. Based on the inter and intra-sample correlation and clustering in PCA, we discarded two outliers (i.e., samples CC-21-065 and CK-21-10). We then performed differential expression analysis of the untransformed read counts using DESeq2 package v1.49.0.^92^ The DESeq2 output was visualized using various R packages (e.g., ggplot2 v4.0.1, ComplexHeatmap v2.26.0, igraph v2.2.1).

#### Acquisition of MS abundance proteomics and phosphoproteomics data for cervical cells stimulated with secretome of HIV-1-infected primary CD4^+^ T cells

Digested samples were analyzed on an Orbitrap Fusion Lumos Tribrid Mass Spectrometer (Thermo Fisher Scientific) equipped with an Easy nLC 1200 ultra-high pressure liquid chromatography system (Thermo Fisher Scientific) or on a timsTOF HT mass spectrometer (Bruker Daltonics) paired with a Vanish Neo UHPLC system (ThermoScientific). For both abundance proteomics and phosphoproteomics analyses, we injected samples on a C18 reverse phase column (25 cm × 75 μm packed with ReprosilPur 1.9-μm particles). We equilibrated analytical columns with 6 μL of mobile phase A with a max pressure of 650 bar. The mobile phase A consisted of 0.1% FA whereas mobile phase B consisted of 0.1% FA / 80% ACN. Peptides were separated by an organic gradient from 4% (2%) to 30% (25%) mobile phase B over 62 minutes followed by an increase to 45% (40%) B over 10 minutes, then held at 95% B for 8 minutes at a flow rate of 300 nL min−1. Data-independent analysis (DIA) was performed on abundance proteomics and phosphoproteomics samples using an 80-minute gradient. An MS scan at 60,000 resolving power over a scan range of 350–1100 *m*/*z*, a normalized AGC target of 300%, and an RF lens setting of 40%. This was followed by DIA scans at 15000 resolving power, using 20 *m*/*z* isolation windows over 350–1100 *m*/*z* at a normalized higher-energy collisional dissociation (HCD) collision energy of 30%. Loop control was set to All. To build a spectral library, one sample from each set of biological replicates was acquired in a data-dependent manner. Data-dependent analysis (DDA) was performed by acquiring a full scan over a *m*/*z* range of 350–1100 in the Orbitrap at 60,000 resolving power with a normalized AGC target of 300% and an RF lens setting of 40%. Dynamic exclusion was set to 45s, with a 10-ppm exclusion width setting. Peptides with charge states 2–6 were selected for MS/MS interrogation using HCD, with 20 MS/MS scans per cycle. The maximum ion injection time was 22 ms. For phosphopeptide-enriched samples, we analyzed MS/MS scans in the Orbitrap using isolation width of 1.6 *m*/*z*, normalized HCD collision energy of 30%, and normalized AGC of 200% at a resolving power of 15,000 with a 22 ms and 40 ms maximum ion injection time for abundance proteomics and phosphoproteomics, respectively.

#### MS abundance proteomics and phosphoproteomics data search

We built experiment specific libraries using mass spectra from each DDA dataset for DIA searches using the Pulsar search engine integrated into Spectronaut v19.8.250311.62635 (Huggins) by searching against a reference Uniprot *Homo sapiens* sequences (downloaded 7 October 2024) and an HIV-1 pNL4-3 proteome of 9 viral proteins, including gag polyprotein, pol polyprotein, vif, vpr, tat, rev, vpu, env polyprotein and nef. For protein abundance samples, data were searched using the default Biognosys (BGS) settings, variable modification of methionine oxidation, static modification of carbamidomethyl cysteine, and filtering to a final 1% FDR at the peptide, peptide spectrum match (PSM) and protein level. For phosphopeptide-enriched samples, BGS settings were modified to include phosphorylation of S, T and Y as a variable modification. We used the generated search libraries to search the DIA data. For protein abundance samples, we used default BGS settings with no data normalization. For phosphopeptide-enriched samples, we applied the PTM site localization score in Spectronaut. Imputation was not performed for all the analyses.

#### Quantitative analysis of MS abundance proteomics and phosphoproteomics datasets from cervical cells

Quantitative analysis and visualization were performed in the R statistical programming language (v4.5.2). For protein abundance data, we utilized a robust R package known as Differential Enrichment analysis of Proteomics data (DEP v1.32.0^93^), which provides an integrated analysis workflow for differential protein expression of MS abundance proteomic data. The DEP workflow has various functions for data preparation, filtering, variance normalization (using variance stabilizing transformation; vsn) and imputation of missing values and statistical testing of differentially expressed proteins between HIV-1-infected and HIV-uninfected conditions using moderated *t*-test from limma v3.65.1.^94^ In our case, we did not impute missing values. We also minimized intra- and inter-sample variability by selecting peptides with coefficient of variation ≤0.3 for DEP workflow.

For phosphoproteomic data, statistical analysis of phosphorylation changes between HIV-1-infected and HIV-uninfected runs were computed using peptide ion fragment data output from Spectronaut. The phosphosites with high confidence localization probability (>0.75) were subjected to Fisher enrichment analysis (“Phosphoproteomics Enrichment Analysis”) in The Kinase Library platform (https://kinase-library.phosphosite.org/kinase-library/fisher-enrichment-analysis; kinase-library v1.5.0).^70^^,^^71^ Kinase activities generated from this analysis were visualized using ggplot2 and ComplexHeatmap packages in R software.

## Supplemental information

Document S1. Figures S1–S6

Data S1. Tables S1–S8 Table S1. Study participants. (A) Participants demographic and clinical characteristics. (B) Participants eligibility questionnaire. (C) Study inclusion and exclusion criteria, related to Figures 1 and 2. Table S2. Network propagation outputs for the top altered TCGA cervical cancer genes size-matched to the RNA-sequencing up genes in HIV-1 infected women with normal cervix, related to Figure 3C. Table S3. Gene set overrepresentation analysis output for six network clusters, related to Figure 3E. Table S4. Network propagation outputs for the top altered TCGA cervical cancer genes size-matched to proteome up in C33A cells treated with HIV-1 infected CD4+ T-cell conditioned media, related to Figure 4C. Table S5. Gene set overrepresentation analysis output for 17 network clusters, related to Figure 4E. Table S6. Gene set overrepresentation analysis output for the size-matched upregulated genes for RNA-sequencing and mass spectrometry analysis, related to Figure 5A. Table S7. Gene set overrepresentation analysis output for two network clusters, related to Figure 5C. Table S8. Datasets used for final visualization. Abundance proteomics (A) and phosphoproteomics (B) data of endogenous C33A cells treated with HIV-infected or uninfected CD4+ T-cell conditioned media. Phosphoproteomics data was filtered for localization probability >0.75. (C) RNA sequencing data of cervical swabs of Kenyan women living with or without HIV stratified based on cancer status.
