# Supplementary material for: Paracrine signals from HIV-1-infected immune cells reprogram cervical cancer pathways
Source: iScience. 2026 Jun 4;29(6):116083. doi: 10.1016/j.isci.2026.116083 (PMC13265902; doi:10.1016/j.isci.2026.116083)
Supplement: Document S1. Figures S1–S6 [file mmc1.pdf]

iScience, Volume *29*

Supplemental information

Paracrine signals from HIV-1-infected immune cells reprogram cervical cancer pathways

Charles Ochieng' Olwal, Ujjwal Rathore, Sara K. Makanani, Prashant Kaushal, Immy A. Ashley, Manisha R. Ummadi, Vincent Appiah, Alexandra Lindsey Djomkam Zune, Sophie F. Blanc, Declan M. Winters, Yennifer Delgado, Kapten Muthoka, Jacqueline M. Fabius, Manon Eckhardt, Robyn M. Kaake, Maureen Su, Oliver I. Fregoso, Judd F. Hultquist, Elkanah Omenge Orang'o, Danielle L. Swaney, George Boateng Kyei, Nevan J. Krogan, Peter Kojo Quashie, Yaw Bediako, and Mehdi Bouhaddou

Figure S1

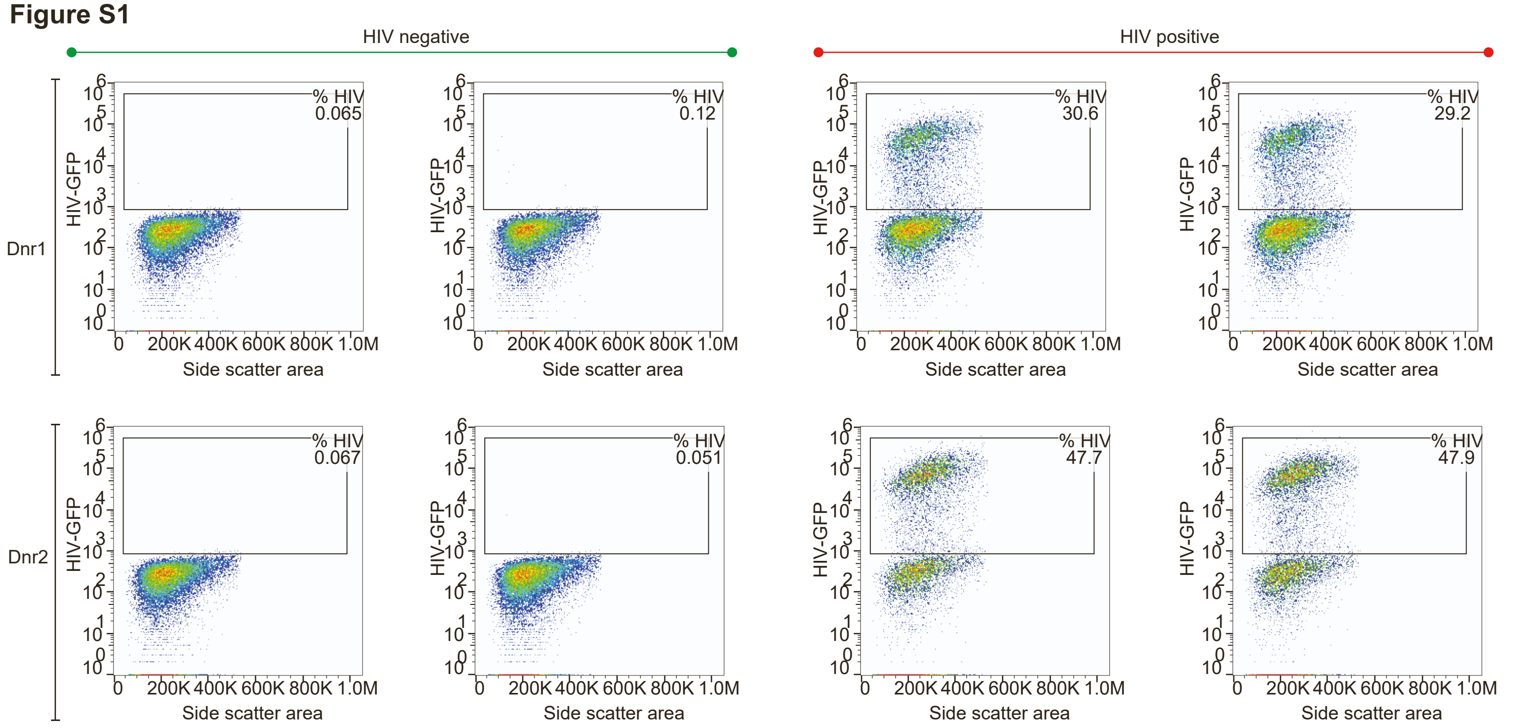

Figure S1. Infection of primary CD+ T-cells with HIV-1. Flow cytometry outputs for primary CD4+ T-cells uninfected (**Left**) or infected with a GFP-tagged replication competent HIV-1 strain (**Right**). The supernatants from these cultures were used to stimulate C33A cells.

Figure S2

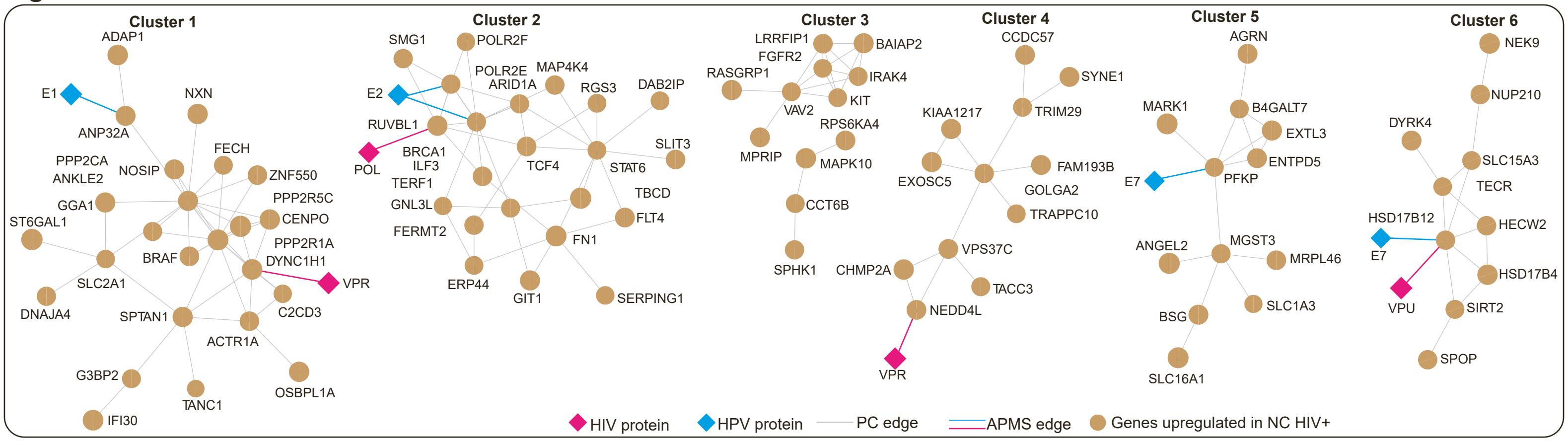

Figure S2. Network sub-clusters composed of differentially upregulated genes from our RNA-seq study and the TCGA Pancancer cervical cancer dataset. The network connections were extracted from Pathway Commons. HIV and HPV protein connections were added to the network based on previous affinity purification mass spectrometry experiments.

Figure S3

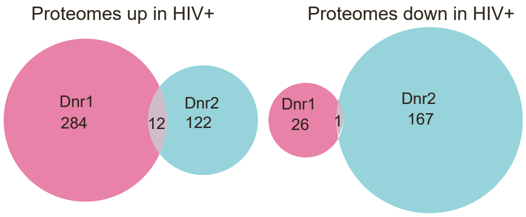

Figure S3. Venn diagrams comparing the number of upregulated (Left) and downregulated (Right) proteins in C33A cells treated with HIV-1 infected CD4+ T-cell conditioned media from two donors. The DEPs were determined based on |log2FC| > 1 and $p < 0.05$ cutoffs.

Figure S4

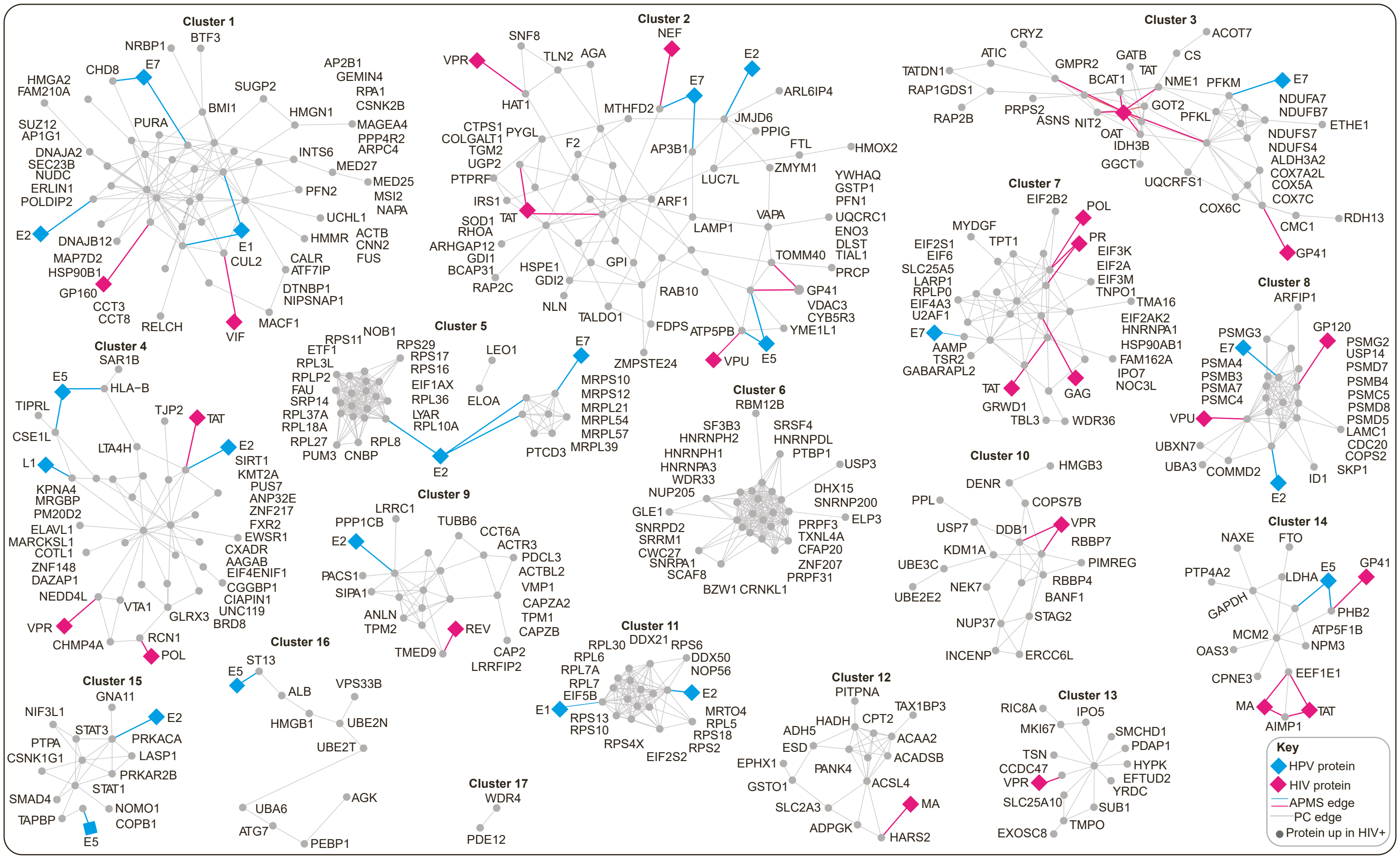

Figure S4. Network depicting interconnection between proteins upregulated in C33A cells exposed to HIV-1 infected CD4+ T-cells conditioned media. All the upregulated proteins from two CD4+ T-cell donors were included. The network connections were extracted from Pathway Commons. HIV and HPV protein connections were added to the network based on previous affinity purification mass spectrometry experiments.

Figure S5

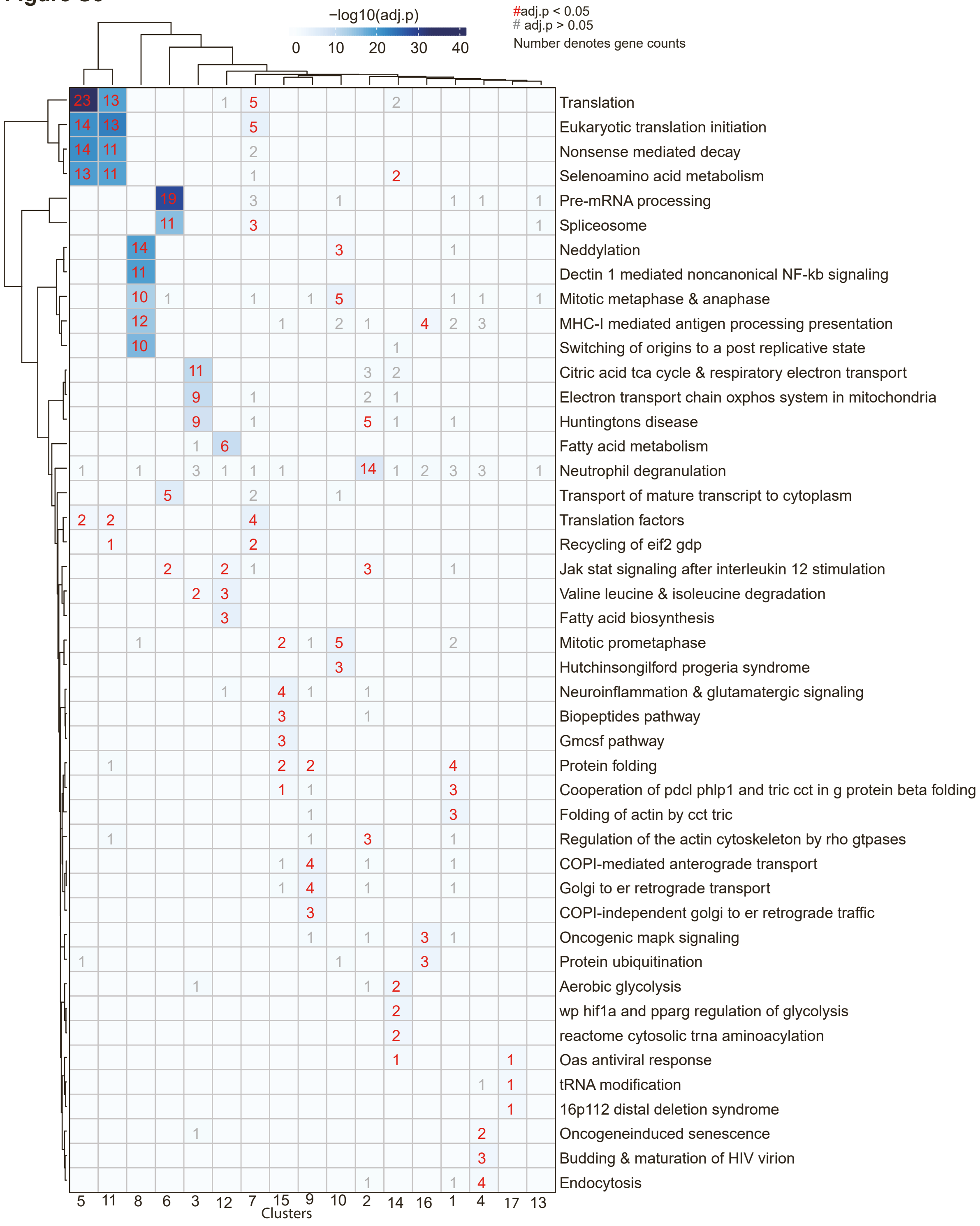

Figure S5. Gene set overrepresentation analysis (GSOA) of subnetworks generated from proteins upregulated in C33A cells treated with HIV-1 infected CD4+ T-cell conditioned media. Enrichment heatmap showing the canonical pathways associated with each of the subnetwork clusters in Figure S3. The GSOA was performed for the network subclusters. The p-values were calculated by hypergeometric test with multiple hypothesis testing correction (false discovery rate; FDR).

Figure S6

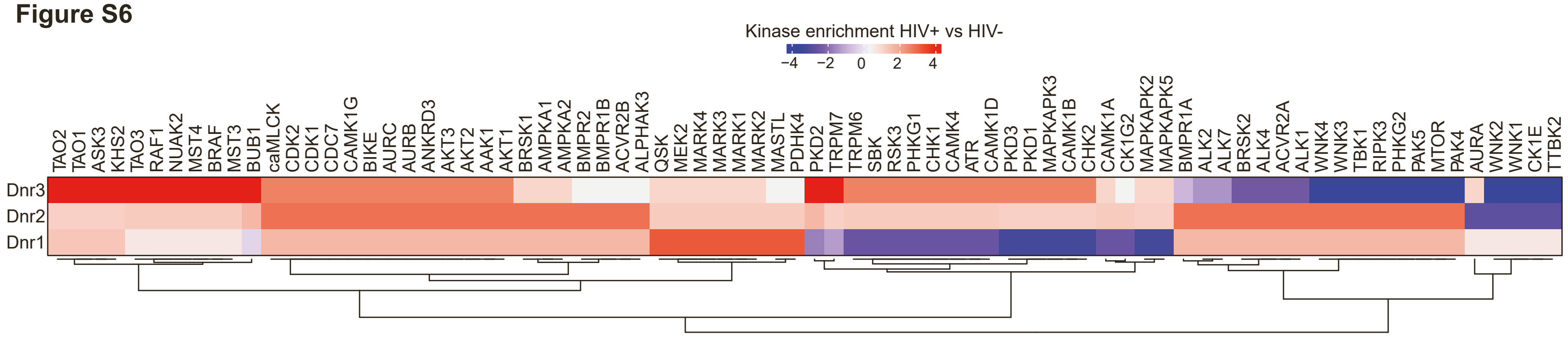

Figure S6. Heatmap showing the activities of top regulated kinases in C33A cells exposed to HIV-1 infected CD4+ T-cells conditioined media isolated from three healthy donors. The top 30 kinases ranked based on the absolute kinase enrichment/activity are visualized. The colors indicate an increase (red) or decrease (blue) in kinase activity.
